# Blockchain and Machine Learning Inspired Secure Smart Home Communication Network

**DOI:** 10.3390/s23136132

**Published:** 2023-07-04

**Authors:** Subhita Menon, Divya Anand, Kavita, Sahil Verma, Manider Kaur, N. Z. Jhanjhi, Rania M. Ghoniem, Sayan Kumar Ray

**Affiliations:** 1 School of Computer Science and Engineering, Lovely Professional University, Phagwara 144411, India; subhitamenon1@gmail.com (S.M.); divya.24844@lpu.co.in (D.A.); 2 Department of Computer Science and Engineering, Uttaranchal University, Dehradun 248007, India; kavita@ieee.org (K.); sahilverma@ieee.org (S.V.); 3 School of Computer Science and Engineering, Guru Gobind Singh College for Women, Chandigarh 160019, India; maninderkaur@ggscw.ac.in; 4 School of Computer Science (SCS), Taylor’s University, Subang Jaya 47500, Malaysia; sayan.ray@taylors.edu.my; 5 Department of Information Technology, College of Computer and Information Sciences, Princess Nourah bint Abdulrahman University, P.O. Box 84428, Riyadh 11671, Saudi Arabia; rmghoniem@pnu.edu.sa

**Keywords:** blockchain, consensus protocol, dragonfly algorithm, Levenberg model, smart contract

## Abstract

With the increasing growth rate of smart home devices and their interconnectivity via the Internet of Things (IoT), security threats to the communication network have become a concern. This paper proposes a learning engine for a smart home communication network that utilizes blockchain-based secure communication and a cloud-based data evaluation layer to segregate and rank data on the basis of three broad categories of Transactions (T), namely Smart T, Mod T, and Avoid T. The learning engine utilizes a neural network for the training and classification of the categories that helps the blockchain layer with improvisation in the decision-making process. The contributions of this paper include the application of a secure blockchain layer for user authentication and the generation of a ledger for the communication network; the utilization of the cloud-based data evaluation layer; the enhancement of an SI-based algorithm for training; and the utilization of a neural engine for the precise training and classification of categories. The proposed algorithm outperformed the Fused Real-Time Sequential Deep Extreme Learning Machine (RTS-DELM) system, the data fusion technique, and artificial intelligence Internet of Things technology in providing electronic information engineering and analyzing optimization schemes in terms of the computation complexity, false authentication rate, and qualitative parameters with a lower average computation complexity; in addition, it ensures a secure, efficient smart home communication network to enhance the lifestyle of human beings.

## 1. Introduction

The concept of the modern world comes with smart technologies that can operate smart homes to enhance the lifestyle of human beings. Smart home devices are connected via Internet of Things (IoT)-based architecture, in which the appliances, specifically smart appliances, are connected to exchange information [1]. The average growth rate of smart homes and their equipment was more than 30%, from 500 million smart home applications to 700 million appliances per year, in the time interval of 2018–2022 [2,3,4,5]. 

**Figure 1:**
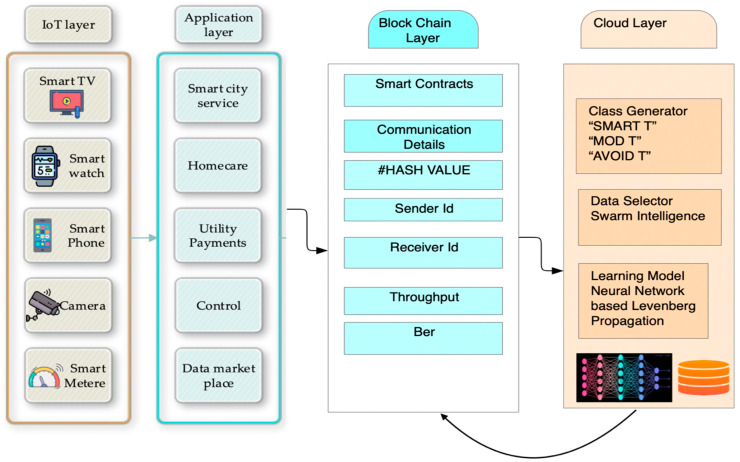
The smart home application architecture.

There are five important aspects related to smart home security and privacy in order to improve the reliability of smart device data transfer. The first is “Authentication”, which helps to verify the communication setup. The second is “Authorization”, which ensures the user’s access rights. The third is “Confidentiality”, which maintains the privacy of data by allowing access to the authorized user. The fourth is “Integration”, which helps minimize data losses and maintains the data in an accurate manner. The fifth is “Availability”, which provides the available service access to authorized users, who are protected from threats. Thus, a smart home network can be sensitive to security threats due to the large number of connected devices [6]. In such cases, a supervised approach to data analysis generated by the IoT network could be quite useful. Swarm intelligence is adequate for solving NP-hard problems. These are mainly used for feature extraction and dimension reduction. Metaheuristic techniques have the potential to address multi-object problems. The population-based metaheuristic algorithm is one of the most effective optimization algorithm architectures. Different types of behaviors are observed in nature, and hence, different algorithms are studied, and the dragonfly algorithm is a powerful data selection tool that can be used to identify patterns and trends in data sets [7]. It is particularly useful for finding outliers and unusual data points. Additionally, some decentralized and deep learning frameworks have also been presented by the research community [8,9,10]. The proposed model represents a significant research contribution to the field of information engineering, as it outperforms existing methods, such as the Fused Real-Time Sequential Deep Extreme Learning Machine (RTS-DELM) system, data fusion techniques, and artificial intelligence Internet of Things technology, in terms of the computation complexity, false authentication rate, and qualitative parameters. With the evolution of blockchain-based communication, the communication data can be kept in a ledger-based record sheet, which is popularly known as a hyperledger in terms of Blockchain [11,12]. This paper focuses on the establishment of a learning engine that is associated with a smart home communication network. To achieve this, a neural-based propagation engine was used for the decision-making of smart transactions, moderate transactions, and to-be-blocked transactions, as shown in Figure 1.

As shown in Figure 1, there are four layers in the application architecture. The first architecture is the IoT layer, in which the users use multiple types of devices and communicate via the application layer. The application layer provides a user Interface (UI) to the user in order to submit and process any request generated by the devices for communication access. The application layer may contain home care, hospitals, city services, data, and marketplaces. This communication generates a bulk amount of data that is aggregated by the blockchain layer. This layer generates the ledger of the communication details and generates a lot of information regarding the transaction that has been made. The transactions are made through Smart Contracts that are established and evaluated in the blockchain layer [13,14,15]. Additionally, the layer creates data in terms of Quality of Service (QoS) parameters, such as Throughput and Bit Error Rate (BER). The blockchain layer is further connected to the Cloud Layer, which stores and processes the data that are generated by the blockchain layer. The cloud layer incorporates the class generator that divides all the data into three segments, namely “SMART T”, “MOD T”, and “AVO-T”, where T represents transactions and MOD and AVO stand for “moderate” and “avoidance”. In the application architecture, the system incorporates a swarm-based data selection algorithm to remove data redundancy, which is further fed to the neural engine for systematic learning. The cloud layer assists the blockchain layer in the decision-making for the transaction. In our article, Random Forest is abbreviated as RF; Naive Bayes is abbreviated as NB; and the proposed result is abbreviated as P. The contributions of the paper are as follows.

(a) The proposed algorithm outperforms the Fused Real-Time Sequential Deep Extreme Learning Machine (RTS-DELM) system, the data fusion technique, and artificial intelligence Internet of Things technology in providing electronic information engineering and analysis optimization schemes in terms of the computation complexity, false authentication rate, and qualitative parameters with a lower average computation complexity; in addition, it ensures a secure, efficient smart home communication network to enhance the lifestyle of human beings.
(b) Application of a secure blockchain layer for user authentication and the generation of a ledger for the communication network.
(c) Application of a cloud-based data evaluation layer to segregate and process data to generate a rank model based on three broad categories of transactions.
(d) Utilization and enhancement of an SI-based algorithm for the preciseness of the training in the cloud layer.
(e) Utilization of a neural engine for the training and classification of the categories that help the blockchain layer with improvisation in the decision-making of the blockchain layer.

The rest of the paper is organized in the following manner. Section 2 illustrates the related work that has been performed in the enhancement of the smart home-based application architecture. Section 3 incorporates the proposed work that supports machine learning (ML), blockchain, and swarm-based application architecture for the enhancement of the smart home network. Section 4 contains the experimental results and discussion, and finally, the paper is concluded in Section 5.

## 2. Related Work

This literature review provides an overview of recent studies on smart home technologies. While conducting the literature review, the authors investigated the concept of peer-to-peer energy trading among smart homes. Peer-to-peer energy represents the communication among the attached devices with routing topology [2]. The next review was on a blockchain-based smart home network security model based on a decentralized network that used fused machine learning. The author’s aim of fusing network security with machine learning was to reuse the calculations and the distributions that were made in the communication between smart devices. The target was to reduce the computation delay and provide security via a cloud platform [3]. The author proposed a data security model for smart homes that used blockchain technology via blockchain interpretation; the security of the overall system was enhanced, as it kept a record of communication and every linked communication. The model introduced an encrypted communication index termed the hash value. The hash was maintained in an open-source ledger for the virtualization of the entire concept; in this, the difficulty target could be used to control the working phase of machines for new block generation [4]. 

The machine learning intelligence approach was evaluated on the basis of energy consumption and was proposed by the author to reduce the expenses of smart homes [16]. It aims to find the resources that are used the least as well as those that are highly used. The authors relied on the training and classification mechanism to provide an understanding of the system [11]. While conducting the review, the author introduced smart home networks developed on the basis of the occupancy detection model using interoperable building automation technologies. The model implemented propagational neural networks for the recognition of occupancies via a detection mechanism. The cloud was utilized for data centralization and monitoring purposes. It is often observed that security is one of the major concerns in blockchain-based IoT frameworks and architecture, along with power and performance efficacy. The communication aspect and the channel modeling through which the data are transferred from one end to another are also an area of discussion in the same context. The efficiency aspect was the main focus of these papers, as they proposed different optimization algorithms to reduce energy consumption and improve the efficiency of smart homes and IoT devices [12]. The author proposed an optimization algorithm based on the bat algorithm with inertia weight to optimize energy consumption and improve the comfort level of smart homes. The proposed algorithm used a feedback mechanism to adjust the parameters of the algorithm and adapt to changes in the environment. The paper mentioned the security measures taken to protect the smart home system from unauthorized access [13].

The author conducted a study on a bio-inspired algorithm called the NBA algorithm, which can optimize energy consumption in wireless sensor networks (WSN) for IoT applications. The algorithm proposed by the author was based on the behavior of honeybees and used a pheromone trail mechanism to guide the search for optimal solutions. The paper mentioned the security measures taken to protect the WSN from attacks, such as jamming and eavesdropping, but it did not discuss them in detail. The paper also proposed a communication protocol based on the message queuing telemetry transport (MQTT) protocol to improve the efficiency of IoT devices [14]. Therefore, the author proposed task management for IoT, and this mechanism was based on predictive optimization to improve the IoT energy efficiency and scalability in smart residential buildings. The proposed mechanism used a machine learning algorithm to predict the energy consumption of IoT devices and schedule their tasks accordingly to reduce energy consumption. The paper briefly mentioned the security measures taken to protect IoT devices from attacks. The paper also proposed a communication protocol based on the Zigbee protocol to improve the efficiency of IoT devices [15]. The contributions and possibilities of the work frame are summarized in Table 1.

The evaluation parameters of the work frame are summarized in Table 2.

## 3. Methodology

The proposed work incorporated the smart home architecture with a machine learning framework that included a Levenberg-based system model for the training and classification of class-generated data. The proposed work is divided into two sections: the blockchain layer and the cloud layer.

### 3.1. The Blockchain Layer

The blockchain layer in the proposed context was utilized to generate the transactions that were made by the user and utility layer. Blockchain is a revolutionary technology that has the potential to revolutionize many industries and sectors. It is a digital ledger that records and verifies transactions, replacing manual processes with automated ones. This makes it easier, faster, and more secure for businesses to complete transactions without relying on a third-party intermediary. In this article, we explore how blockchain technology is used to facilitate transactions across multiple industries, including finance, healthcare, the public sector, and more. We discuss the various benefits of using blockchain in everyday life and provide an overview of the different types of blockchains available today. Finally, we outline some key tips on how to begin using blockchains for your business [18,19]. In a blockchain transaction, each block is chained to the previous block, creating a permanent and public record of all transactions. This allows for secure and transparent transactions, as each block contains a timestamp and unique hash that can be verified by the network. Blockchain technology has the potential to revolutionize the way we conduct business and interact with the world. By creating a secure and transparent record of all transactions, blockchain could help to reduce fraud, improve efficiency, and create new opportunities for businesses and individual. A hash generator is a mathematical function that takes an input of any size and produces an output of a fixed size. The most common type of hash function is the cryptographic hash function, which is used in security applications to protect data from tampering. Cryptographic hash functions are one-way functions, meaning that it is mathematically infeasible to reverse the function and obtain the original input [20]. This makes them ideal for storing data in a secure manner, as any attempt to change the data results in a different hash value being generated. There are many different types of cryptographic hash functions, but the most popular ones are SHA-1 and SHA-256. These functions are used by thousands of websites and applications to protect data and are part of the reason why blockchains are so secure.

The proposed blockchain layer was implemented using the Firebase blockchain platform to host the data. The architecture of the blockchain in Firebase was as follows. Firebase is a cloud-based platform that provides various tools and services for app development. It offers several features, such as real-time databases, cloud storage, authentication, and hosting. In order to use these features in a web or mobile application, the application needs to be configured with a Firebase project [21,22]. The configuration information is stored in a JavaScript object, commonly known as a firebaseConfig object. The given code represents the configuration details of a Firebase project. The firebaseConfig object contains several properties, such as apiKey, authDomain, databaseURL, projectId, storageBucket, appId, and measurementId. These properties hold unique values that are specific to a particular Firebase project. These values are used by the Firebase SDKs to establish a connection between the app and the Firebase services [23,24]. The pseudo-code represents the steps to initialize the firebaseConfig object and set the values for its properties.

1. The firebaseConfig object was initialized with the following properties:
  - apiKey
  - authDomain
  - databaseURL
  - projectId
  - storageBucket
  - appId
  - measurementId
2. The value of the apiKey property was set to “AIzaSyD11_9I9_5yLJu0UCGa6SdiYFQTAiWoOe0”.
3. The value of the authDomain property was set to “subhita-block-chain.firebaseapp.com”.
4. The value of the databaseURL property was set to “https://subhita-block-chain-default-rtdb.firebaseio.com” (accessed on 14 April 2023).
5. The value of the projectId property was set to “subhita-block-chain”.
6. The value of the storageBucket property was set to “subhita-block-chain.appspot.com”.
7. The value of the appId property was set to “1:1038363801308:web:338b2f2XXXX”.
8. The value of the measurementId property was set to “G-38V0GXXXX”.

### 3.2. The Cloud Layer

The cloud layer is made of up three subsections: data separation, data selection, and decision-making with training and classification. The subsections are illustrated below.

#### 3.2.1. Data Separation

**Figure 2:**
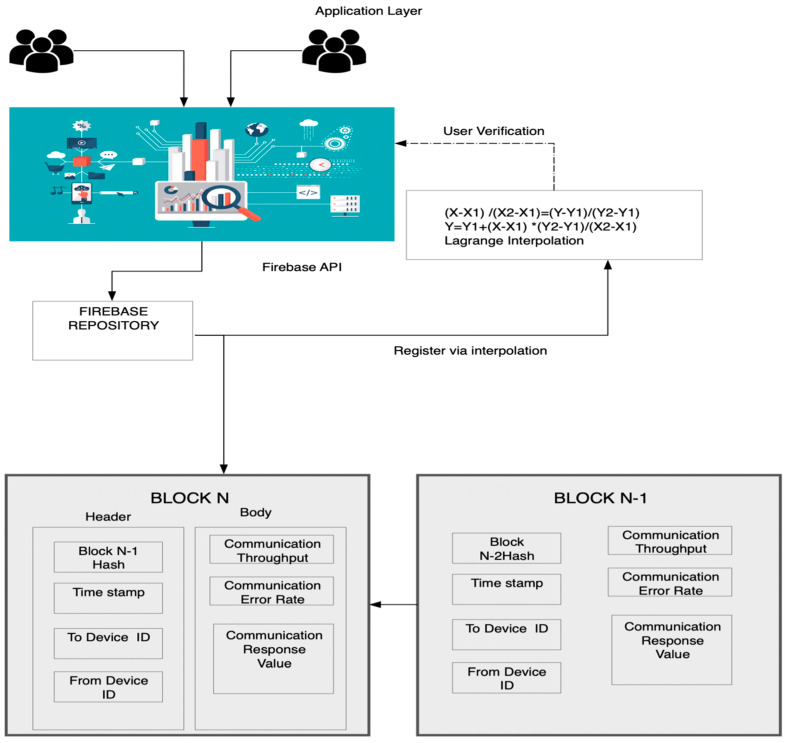
The blockchain layer and generation.

The blockchain layer aggregates the data in the form of a ledger, as illustrated in Section 3.1. The proposed work used the K-means clustering algorithm followed by statistical analysis of the separated data to label them as classes. This section is represented in Figure 2.

The collected data were separated into three classes, whereas the naming convention was performed using Fuzzy Logic. Algorithm 1 illustrates the architecture of the data separation.


**Algorithm 1: Data Separation Algorithm**

Apply data separationInputs: Aggregated Data as AdStart[K_index_, K_cent_] = kmeans(Ad,3)[MSE_K_, STD_K_] = Evaluated_MSE − STD(Ad,K_Index_)Initialize Mamdani Fuzzy Rule SetGT = Label(K_index)End Algorithm


**Figure 3:**
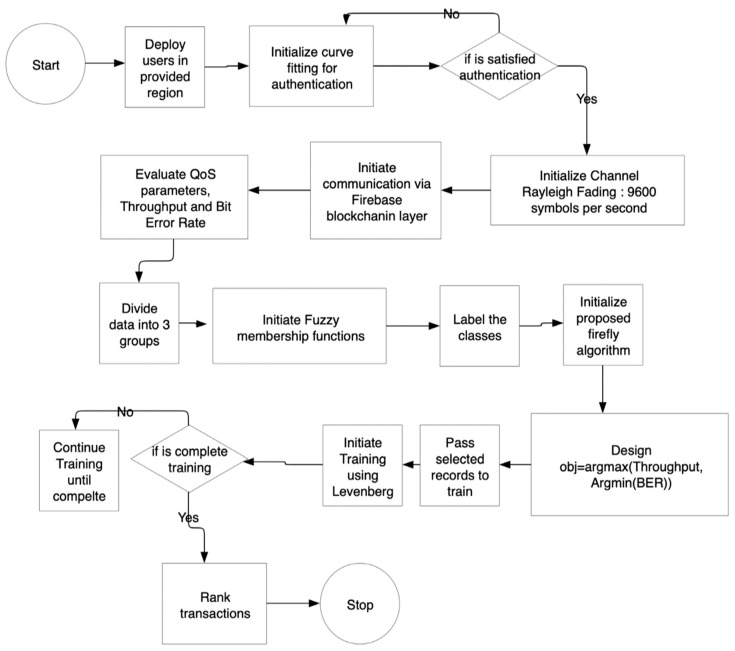
Proposed work model.

Algorithm 1 took all the aggregated data as the input and, as illustrated earlier, divided all the data into 3 groups. The overall work model is presented in Figure 3.

#### 3.2.2. The Data Selection for Ranking

The fuzzy inference engine provided a labeled set based on the evaluated QoS services of the transactions in the blockchain layer. In order to generate a reputation system to assist the blockchain layer, the proposed work utilized the dragonfly algorithm in order to select a data record against its class label. To do so, the proposed work utilized the dragonfly algorithm based on the studied literature that was presented in Section 2.

The dragonfly algorithm is a powerful data selection tool that can be used to identify patterns and trends in data sets. It is particularly useful for finding outliers and unusual data points. The algorithm works by partitioning the data set into small subsets and then iteratively selecting the best subset of data points from each subset. The dragonfly algorithm is fast and efficient, and it can be applied to any type of data set [25,26]. The dragonfly algorithm is a swarm-based algorithm that uses the attraction index for the selection and rejection of the prey. This method is based on the principle of selecting the best data points by considering their distance from the center of the data set. This can be achieved by either using a Euclidean distance or a Manhattan distance [27]. The dragonfly algorithm is able to handle both types of distances, and it also has the ability to consider multiple dimensions when making its calculations [28].

The AI can be calculated using Equation (1). The fuzzy inference engine provides a labeled set based on the evaluated QoS services of the transactions in the blockchain layer. In order to generate a reputation system to assist the blockchain layer, the proposed work utilized the dragonfly algorithm in order to select a data record against its class label. To do so, the proposed work utilized the dragonfly algorithm based on the existing literature [29] to compute the next state of the fly using Equation (1):

(1) $$x_{i}^{t+1}=x_{i}^{t}+\beta_{0}e^{-\gamma r_{ij}^{2}}\left(x_{j}^{t}-x_{i}^{t}\right)+\propto$$

where $x_{i}^{t+1}$ is the next state of the firefly, $x_{i}^{t}$ is the current state of the firefly, ∝ is the randomization parameter {0–1}, r_ij_ is the distance of the distribution x_i_ − x_j_h; here, i and j are the fireflies, β is the attractive index, and γ is the change in the attraction index. Here, x_g_c^t^ is the group centroid of the labeled route. The intended dragonfly algorithms is illustrated in Algorithm 2.


**Algorithm 2: Dragonfly Algorithm**

Dragonflies = [K_index_,Ad]Dragonflies_AK_ = Dragonflies.QoS // Extract QoS parameters for each extracted classG = 10; // Maximum Generation

${\mathrm{Dragonfly}}_{{\mathrm{Score}}_{\mathrm{Chart}}}=\;\mathrm{Zeros}(V,G)\;where\;V\;is\;total\;number\;of\;dragons,\;G\;is\;maximum\;generation$



While G



For i=1:Dragonflies




$S_{t_{p}}={Rand}_{index}\;(Dragonflies,30\%)\;//\;Generate\;a\;30\%\;random\;swarm\;population\;to\;pair$



$S_{p}=Dragonflies[S_{t_{p}}];\;//\;Extract\;the\;population\;attribute\;set$



$For\;j=1:S_{t_{p}}$



$x_{i}^{t}=x_{i}^{t}.QoS\;//\;Extract\;attained\;QoS$



$x_{j}^{t}=x_{j}^{t}.QoS\;//\;Extract\;attained\;QoS$



${AI}_{L}=Evaluate\;attraction\;value\;using\;for\;local\;swarm$



${AI}_{G}=Evaluate\;group\;attraction\;value\;using\;for\;global\;swarm$

$AI=\frac{\left|{AI}_{L}+{AI}_{G}\right|}{2}\;Evaluate\;the\;attraction\;index\;by\;taking$ mean of local and global group

${Dragonfly}_{{Score}_{Chart}}\left(i,j\right)=AI$


End
End


The selected dragonflies were passed to the Neural Engine for the purpose of ranking on the basis of their score generated in the training section. After the completion of the training 30% of all the data underwent rigorous testing in which each identity was tested at least once. Once the data were classified, their classification scores were treated as the rank of the transition. A ground truth value found close to the original value in each case is observed as better option. Neural networks are a type of machine learning algorithm inspired by the structure and function of the human brain. They are used in a variety of applications, from image and speech recognition to fraud detection and financial forecasting. In recent years, neural networks have been applied to the field of blockchain to enhance security in smart homes. The training and classification of blockchain comprise three security categories, namely Smart T, Mod T, and Avoid T for smart homes, which can be achieved through a neural network. The neural network is trained using a dataset of examples, and each example is labeled as either Smart T, Mod T, or Avoid T. The neural network learns to recognize patterns in the data that are associated with each of the three categories and then uses this knowledge to classify new examples [30,31]. The process of training a neural network involves adjusting the weights of its connections between neurons in order to minimize the loss function. The loss function measures the difference between the predicted output of the neural network and the true output. The goal of training is to minimize this difference, which is achieved by adjusting the weights of the connections [32,33,34]. This is achieved using an optimization algorithm, such as stochastic gradient descent, which adjusts the weights in the direction that reduces the loss function the most [35,36]. Once the neural network has been trained, it can be used for classification.

**Figure 4:**
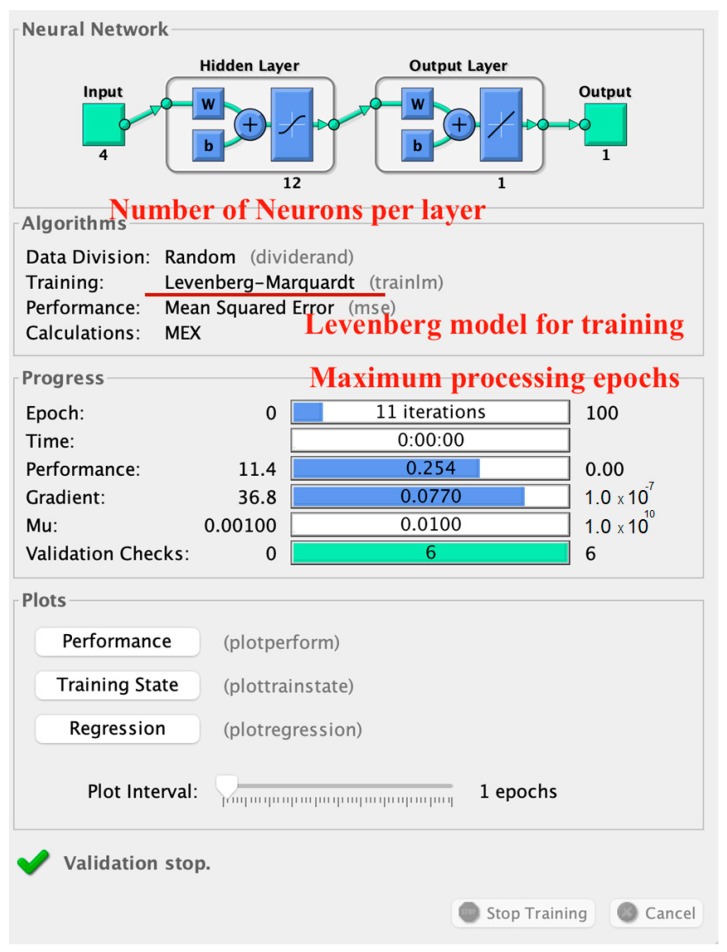
Neural network structure.

Figure 4 illustrates that the proposed work utilized four parameters, namely the sender id, the receiver id, the evaluated Throughput, and BER, as the input for the Levenberg model, in which the total number of supplied layers in the model was determined to be two, and there were a total of 12 active neurons per layer. The neurons were propagated for the minimum gradient, which was the Mean Squared Error in the case of the proposed work. The total number of supplied epochs per iteration was 100, and the gradient is attained between 5–15 epochs on average. The ground truth values for the data records were the class labels generated by the fuzzy inference engine. To classify a new example, the input was fed into the neural network, which generated an output. This output was a probability distribution over the three categories, indicating the probability that the input belonged to each category. The category with the highest probability was chosen as the classification. The proposed work was evaluated on the basis of QoS and the quantitative parameters that are illustrated in the next section.

## 4. Results and Discussion

The proposed model of a smart home network was designed in the Matlab Simulation and Google Colab, and the blockchain ledger was created to evaluate the effects of QoS and quantitative parameters, namely the overall computation complexity, false detection rate, precision, recall, and f-measure, along with classification accuracy. In order to do so, the proposed work performed 20,000 simulations with the following system model to collect the Throughput and BER of each simulation, which were fed to the learning mechanism. The proposed work was tested for more than 1000 iterations, and on average, the convergence of the global optimum was found to be close to the global optimum.

The system specifications with reference to blockchain and cloud computing technology are summarized in Table 3. Additionally, the system and system model specifications are stated in Table 4.

The proposed work was compared with two state-of-the-art algorithms in terms of QoS and evaluated for multiple classifiers with quantitative parameters. The training and classification model was used on the transaction record and categorized it by providing a label of Smart T, Mod T, or Avoid T. The neural network learns to recognize patterns in the data that are associated with each of the three categories and then uses this knowledge to classify new examples. The computation complexity can increase significantly as the number of devices and transactions in the network increases. Additionally, in a proof-of-work (PoW) blockchain, the process of mining can be very computationally intensive, which can be a challenge for resource-constrained IoT devices.

Table 5 presents the value of the computation complexity and the comparison with other state-of-the-art techniques for similar types of environments.

**Figure 5:**
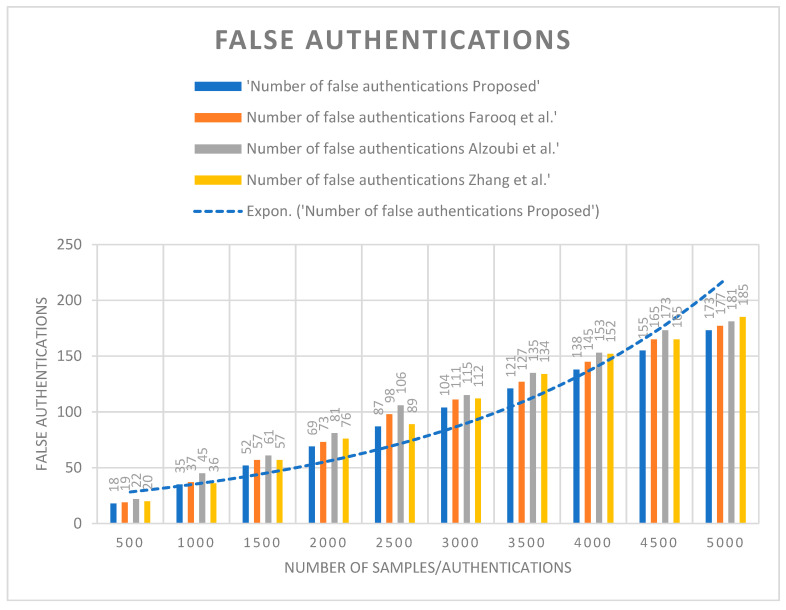
Number of false authentications against Farooq et al. [3], Alzoubi et al. [11], and Zhang et al. [21].

The average computation complexity of the proposed algorithm across all test samples was lower than that of the other algorithms. This can be seen by comparing the average value of the ‘Computation Complexity Proposed’ column to the average values of the other columns. For example, the average computation complexity of the proposed algorithm for all test samples was 3.544, while the average computation complexity of the algorithm based on blockchain and fused machine learning was 3.944, indicating an improvement of 10.14% by the proposed algorithm over that in the existing study. The percent improvement of the proposed algorithm over the other algorithms varied depending on the number of test samples. For example, for 500 test samples, the proposed algorithm had a computation complexity of 0.765, which was 11.1% lower. However, for 5000 test samples, the percent improvement over the existing study dropped to 7.07%. The reason for the improved performance of the proposed algorithm is attributed to its efficient data selection, as indicated in the table by the decreasing values of proposed computation complexity with increasing test samples. The proposed work was also evaluated for the false authentication rate against the same set of algorithms. The authentication rate in Figure 5 represents the total number of false detections while applying authentication over the users.

The proposed algorithm showed lower numbers of false authentications compared with the other algorithms. This was reflected in the % improvement values, which show the percent reduction in false authentications compared with each of the other algorithms. For example, in a total test sample size of 500, the proposed algorithm had 18 false authentications, while the next-best algorithm had 19 false authentications. This represents a % improvement of 5.26% for the proposed algorithm. Similarly, in a total test sample size of 5000, the proposed algorithm had 173 false authentications, while the other had 185 false authentications. This represents a % improvement of 6.49% for the proposed algorithm. As illustrated in this section, the proposed work was also evaluated for qualitative parameters. Table 6 presents the qualitative analysis.

**Figure 6:**
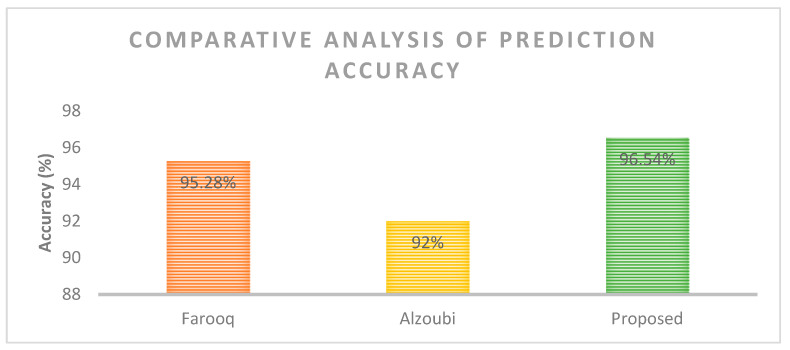
Accuracy comparison.

In terms of precision, the proposed method performed consistently well, with an average precision of around 0.97–0.98. The NB and RF methods had similar precision scores for most cases, but the RF method had lower precision for some test sample sizes, particularly for larger test samples. For recall, the proposed method performed well, with an average score of around 0.98–0.99. The NB and RF methods also performed well but with slightly lower scores than the proposed method. In terms of the F-measure, the proposed method again performed consistently well, with an average score of around 0.96–0.99. The NB and RF methods had similar F-measure scores in most cases, but the RF method had a lower F-measure for some test sample sizes, particularly for larger test samples. The performance of the proposed work was further compared for the prediction accuracy of false authentications. Figure 6 shows the comparative analysis of the proposed work performed against the existing works in terms of the prediction of false authentications. It was observed that the proposed work exhibited a high accuracy of 96.54%, which is a little higher than that exhibited by Farooq (95.28%) and Alzoubi (92%), thus justifying the effectiveness of the proposed work in smart home networks.

The proposed work reduced the computation complexity in terms of overall computation, which will help to transfer more data in the network. The network is capable of running other real-time datasets as well if they are designated to work on a similar aspect.

## 5. Conclusions

This research paper proposed an architecture that incorporates IoT, blockchain, cloud, and artificial intelligence techniques for a secure and efficient communication network. The paper provides a detailed description of the proposed architecture and its components, including the user interface, ledger generation, data evaluation, and decision-making techniques. The contributions of this paper are the secure application of the blockchain layer and cloud-based data evaluation layer and the efficient utilization of the artificial intelligence-based algorithm. In order to incorporate efficient communication and security aspects, the paper designed an updated dragonfly algorithm that supports the training layer to pick the best samples for the three classes designed, namely “SMART T”, “MOD-T”, and “AVO-T”. Specifically, for all test samples, the proposed algorithm had an average computation complexity of 3.544, while the existing algorithm had an average computation complexity of 3.944, indicating an improvement of 10.14% by the proposed algorithm. The percent improvement varied depending on the number of test samples, with the proposed algorithm showing a lower computation complexity than the existing algorithm based on blockchain and fused machine learning for all test sample sizes but with a decreasing percent improvement as the number of test samples increased. The improved performance of the proposed algorithm is attributed to its efficient data selection, which is indicated by the decreasing values of proposed computation complexity with increasing test samples. In the case of false authentication analysis for a sample size of 5000, the proposed algorithm had 173 false authentications, while the existing work of Farooq, Alzoubi, and Zhang had 177, 181, and 185 false authentications, respectively. This represents a maximum % improvement of 6.49% for the proposed algorithm. Furthermore, the proposed work exhibited a high prediction accuracy of 96.54% for identifying false authentications, thus proving the ability of the proposed work to handle security in smart home networks in comparison with existing works. Furthermore, this research provides future possibilities for the integration of these techniques with other technologies, such as 5G and edge computing, for more secure and efficient communication networks. Overall, the proposed architecture has potential applications in various fields, such as home care, hospitals, city services, and data marketplaces.

## Figures and Tables

**Table 1:** Comparative analysis of existing methodologies.

Author/Citation	Implemented Technology	Methodology	Findings
Alam et al. (2019) [2]	Smart home	Peer-to-peer energy trading with routing topology	Cost saving is not directly proportional to the increase in the usage of renewable resources
Tchagna Kouanou et al. (2022) [5]	Smart home data security using Blockchain technology	Blockchain interpretation was used to keep communication records	Enhancing security of the overall system via encrypted communication index
Mansouri et al. (2023) [8]	Hierarchical decentralized framework technology	Deep learning-based forecaster and risk-aware information gap decision theory were employed to design efficient smart homes	The smart prosumer’s illustrated reduced energy requirements
Alzoubi et al. (2022) [11]	Machine learning technology	Involved bat optimization technique for reduced energy consumption of the smart home	Using machine learning for intelligent evaluation of energy consumption and finding the least used and highly used resources
Rivera et al. (2015) [12]	Automation technology	Built smart homes using interoperable automation using neural networks	Designed efficient smart homes with reduced energy consumption
Vanus et al. (2022) [17]	Occupancy detection for smart homes	Propagational neural networks	Developed an occupancy detection model and utilized the cloud for data centralization and monitoring purposes

**Table 2:** Evaluation parameters.

Author	Statistical Measure	Number of Collected Packets	File Size (Bytes)	Amount of Data Collected (Bytes)	Average Data Transfer Rate (B/s)
Alam et al. (2019) [2]	Mean	4958	8,318,400	4,119,314	4.1
Vanus et al. (2022) [17]	Median	2048	1,200,000	1,167,360	6.5
Qamar et al. (2022) [13]	Median	1000	1,048,576	982,254	6.5
Kumar et al. (2022) [14]	Mean	50	10,111	3424	0.5
Khanpara et al. (2023) [15]	Mean	15,360	1,094,430	748,408	4.5
Malek et al. (2022) [18]	Mean	1000	32,000	15,814	6.2
Devassy et al. (2022) [19]	Mean	100	15,423	4846	0.6

**Table 3:** System specifications.

Number of data centers	2
Number of PMs to handle	10
Cloud Type	Xen
Blockchain Type	Firebase

**Table 4:** Ordinal measures of system and system model.

**System Information**	
RAM	4 GB
Processor	intel core i3 530
Memory	DDR3
HD Capacity	500 GB
**System Model Information**	
Number of Users	50–500
Simulation Area	4000 m^2^
Channel of Communication	Rayleigh channel
Interpolation measurement	Lagrange interpolation
Channel Capacity	96,000 symbols per second
Channel gain	0.0023 units
Evaluation Parameters	Throughput, BER

**Table 5:** Computation complexity.

Total Test Samples	Fused Real-Time Sequential Deep Extreme Learning Machine System [3]	Data Fusion Technique [11]	Internet of Things for Electronic Information Engineering and Optimization Schemes [17]	Proposed Technique
500	0.85991282	0.8780829	0.84910773	0.76528091
1000	2.11021973	1.9531654	2.04723132	1.87965372
1500	1.78525512	1.7024945	1.82948338	1.67876619
2000	2.05459249	2.1310947	2.19553898	2.03360384
2500	3.74510098	3.6378217	3.52360793	3.46864933
3000	3.34175878	3.4041558	3.48210032	3.26647149
3500	4.86868341	5.0819671	4.60406431	4.44362262
4000	4.22346545	4.2047017	4.47550357	4.06799277
4500	4.91378696	4.7681754	5.12593208	4.50671531
5000	6.0492668	6.4493703	6.17813064	5.77015973

**Table 6:** Quantitative parameter evaluation.

	Precision			Recall			F-Measure		
Total Test Samples	RF	NB	P	RF	NB	P	RF	NB	P
500	0.98	0.98	0.98	0.89	0.97	0.97	0.93	0.97	0.98
1000	0.97	0.97	0.97	0.93	0.99	0.99	0.95	0.98	0.98
1500	0.96	0.96	0.97	0.94	0.96	0.96	0.95	0.96	0.96
2000	0.97	0.97	0.97	0.93	0.99	0.99	0.95	0.98	0.98
2500	0.96	0.95	0.96	0.99	0.99	0.99	0.97	0.97	0.97
3000	0.98	0.98	0.98	0.94	1	1	0.96	0.99	0.99
3500	0.97	0.97	0.97	0.91	0.99	0.99	0.94	0.98	0.98
4000	0.98	0.98	0.99	0.95	0.98	0.98	0.96	0.98	0.98
4500	0.95	0.95	0.96	0.95	0.99	0.99	0.95	0.97	0.97
5000	0.93	0.96	0.98	0.93	0.92	0.95	0.93	0.92	0.97
